# Impact on health‐related quality of life deterioration‐free survival of a first‐line therapy combining nab‐paclitaxel plus either gemcitabine or simplified leucovorin and fluorouracil for patients with metastatic pancreatic cancer: Results of the randomized phase II AFUGEM GERCOR clinical trial

**DOI:** 10.1002/cam4.2311

**Published:** 2019-07-17

**Authors:** Emilie Charton, Jean‐Baptiste Bachet, Pascal Hammel, Jérôme Desramé, Benoist Chibaudel, Romain Cohen, Philippe Debourdeau, Jérome Dauba, Thierry Lecomte, Jean‐François Seitz, Christophe Tournigand, Thomas Aparicio, Véronique Guerin‐Meyer, Julien Taieb, Julien Volet, Christophe Louvet, Amélie Anota, Franck Bonnetain

**Affiliations:** ^1^ Methodology and Quality of Life Unit in Oncology INSERM UMR 1098 University Hospital of Besançon Besançon France; ^2^ University Bourgogne Franche‐Comté, INSERM, EFS BFC, UMR1098, Interactions Hôte‐Greffon‐Tumeur/Ingénierie Cellulaire et Génique Besançon France; ^3^ Department of Hepato‐Gastroenterology, Groupe hospitalier Pitié Salpêtrière Sorbonne University, UPMC University Paris France; ^4^ Department of Digestive Oncology Hôpital Beaujon Clichy France; ^5^ Department of Hepato‐Gastroenterology Hôpital Privé Jean Mermoz Lyon France; ^6^ Department of Oncology Institut Franco‐Britannique Levallois‐Perret France; ^7^ Department of Oncology AP‐HP, Hôpital Saint‐Antoine, Sorbonne University Paris France; ^8^ Department of Oncology Institut Saint Catherine Avignon France; ^9^ Department of Oncology Hôpital Layne Mont de Marsan Mont de Marsan France; ^10^ Department of Hepato‐Gastroenterology Hôpital Trousseau Tours France; ^11^ CHU La Timone Marseille France; ^12^ Department of Oncology CHU Henri Mondor Créteil France; ^13^ Department of Hepato‐Gastroenterology CHU Saint Louis Paris France; ^14^ Department of Oncology Institut de cancérologie de L'Ouest Paul Papin Angers France; ^15^ Department of Gastroenterology and Digestive Oncology Hôpital Européen Georges Pompidou Paris France; ^16^ Department of Hepato‐Gastroenterology CHU Robert Debré Reims France; ^17^ Department of Oncology Institut Mutualiste Montsouris Paris France; ^18^ French National Platform of Quality of Life and Cancer Besançon France

**Keywords:** clinical trial, deterioration‐free survival, metastatic, pancreatic cancer, quality of life

## Abstract

**Background:**

The phase II AFUGEM GERCOR trial aimed to assess the efficacy of a first‐line therapy combining nab‐paclitaxel plus either gemcitabine (gemcitabine group) or simplified leucovorin and fluorouracil (sLV5FU2 group) in patients with previously untreated metastatic pancreatic cancer. Results of progression‐free survival at 4 months (primary endpoint) were in favor of the sLV5FU2 group. This paper presents health‐related quality of life (HRQoL) data as a secondary endpoint.

**Methods:**

HRQoL was assessed using the EORTC QLQ‐C30 questionnaire at baseline and at each chemotherapy cycle until the end of treatment. The HRQoL deterioration‐free survival (QFS) was used as a modality of longitudinal analysis. QFS was defined as the time between randomization and the first definitive HRQoL score deterioration as compared to the baseline score, or death. Sensitivity analysis was performed excluding death as an event. Univariate Cox models were used to estimate hazard ratios (HRs) and 90% confidence intervals (CIs) of the treatment effect.

**Results:**

Between 2013 and 2014, 114 patients were randomized in a 1:2 ratio (39 in the gemcitabine group and 75 in the sLV5FU2 group). Patients in the sLV5FU2 group seemed to present longer QFS than those of the gemcitabine group for 14 out of 15 dimensions, with HRs < 1. Results of the sensitivity analysis excluding death as an event were significantly in favor of the sLV5FU2 group for physical functioning (HR = 0.51 [90% CI 0.27‐0.97]) and pain (HR = 0.26 [90% CI 0.09‐0.74]).

**Conclusion:**

The nab‐paclitaxel plus simplified leucovorin and fluorouracil combination had no negative impact in exploratory HRQoL analyses.

## 1 INTRODUCTION

Pancreatic cancer is a devastating disease with an overall 5‐year survival of less than 5%.^1^, ^2^ The mortality trend is increasing in both genders,^3^, ^4^ and pancreatic cancer is one of the most common causes of death from cancer.^5^

Over the past two decades, gemcitabine monotherapy has been a standard treatment for metastatic pancreatic cancer.^6^ In 2011, the FOLFIRINOX regimen (fluorouracil, leucovorin, irinotecan, and oxaliplatin)^7^, ^8^ and the combination of gemcitabine with nab‐paclitaxel^9^, ^10^ demonstrated an improvement in progression‐free survival and overall survival compared with gemcitabine alone. These regimens are thus now considered as the standard first‐line treatment options in patients with metastatic pancreatic cancer and good general status without marked cholestasis.

In the randomized, phase II, AFUGEM GERCOR (Groupe Coopérateur Multidisciplinaire en Oncologie) trial, nab‐paclitaxel plus simplified leucovorin and fluorouracil treatment (sLV5FU2 group) improved the primary endpoint of progression‐free survival at 4 months in the first 72 assessable patients in the sLV5FU2 group, and the secondary endpoint of overall survival compared to nab‐paclitaxel plus gemcitabine treatment (gemcitabine group).^11^ At 4 months, 40 (56% [90% confidence interval (CI) 45‐66]) out of 72 patients in the sLV5FU2 group were alive and free of disease progression vs 21 (54% [90% CI 40‐68]) out of 39 patients in the gemcitabine group. In exploratory analyses, the median progression‐free survival was 5.9 months [95% CI 3.6‐7.4] in the sLV5FU2 group vs 4.9 months [95% CI 2.1‐7.7] in the gemcitabine group. Similarly, the median overall survival was 11.4 months [95% CI 8.8‐16.5] in the sLV5FU2 group vs 9.2 months [95% CI 6.0‐13.6] in the gemcitabine group (exploratory hazard ratio (HR) of 0.61 [95% CI 0.40‐0.95]). Although these results appear to be promising for the nab‐paclitaxel plus sLV5FU2 combination, it is crucial to study the impact of the treatment on patients’ health‐related quality of life (HRQoL) over time. In fact, new combinations of drugs can cause adverse events that may deteriorate the patients’ perception of their health. Thus, it is particularly important to take the patient's HRQoL level into account in disease management, in order to ensure that the new treatment does not yield a clinical benefit at the cost of reduced quality of life.

In this context, based on the phase II AFUGEM clinical trial, we report the impact on HRQoL of a first‐line therapy combining nab‐paclitaxel plus either gemcitabine or sLV5FU2 in patients with previously untreated metastatic pancreatic cancer.

## 2 METHODS

### 2.1 Patients and eligibility criteria

The AFUGEM study was an open‐label, noncomparative, randomized, multicentre, phase II clinical trial, conducted in 15 centers in France (ClinicalTrials.gov number NCT01964534).

Eligible patients were required to be aged at least 18 years, with histologically or cytologically proven adenocarcinoma of the pancreas, stage IV disease, no prior therapy for metastatic disease, an Eastern Cooperative Oncology Group (ECOG) performance status 0‐2, and presenting adequate hematologic, renal, and liver function. The detailed eligibility criteria have previously been reported.^12^ The protocol was approved by the French Ethics Committee and written informed consent was obtained from all patients before randomization.

Using a minimization technique stratified by center and ECOG performance status, patients were randomly assigned (1:2 ratio) to receive nab‐paclitaxel plus gemcitabine (control arm) or nab‐paclitaxel plus sLV5FU2 (experimental arm). Both regimens were administered every 28 days and details of the regimens have previously been published.^12^

The primary endpoint was progression‐free survival at 4 months in the first 72 patients in the sLV5FU2 group. Secondary endpoints were objective response, progression‐free survival, overall survival, tolerance and HRQoL.

### 2.2 Health‐related quality of life assessment

HRQoL was assessed in each treatment arm using the European Organisation for Research and Treatment of Cancer Quality of Life Questionnaire C30 (EORTC QLQ‐C30) cancer‐specific questionnaire,^13^ at baseline and at each chemotherapy cycle until the end of treatment. The QLQ‐C30 includes 30 items and assesses global health status, 5 functional scales (physical, role, emotional, cognitive and social functioning) and 9 symptom scales (fatigue, nausea and vomiting, pain, dyspnoea, insomnia, appetite loss, constipation, diarrhea, and financial difficulties). Scores vary from 0 (worst) to 100 (best) for global health status and functional scales, and from 0 (best) to 100 (worst) for the symptom scales.

### 2.3 Statistical analysis

#### 2.3.1 Population and statistical considerations

The intention‐to‐treat (ITT) population was considered in the HRQoL analysis, that is, all randomized patients regardless of their eligibility criteria and treatment received. Due to the occurrence of missing data, a modified ITT (mITT) population was also defined as all ITT patients with at least one HRQoL score available at baseline.^14^

Five targeted dimensions were defined a priori in the protocol: physical functioning, emotional functioning, fatigue, pain, and appetite loss. Other dimensions were regarded as being exploratory.^12^

Although HRQoL was a secondary endpoint in this study, a decision‐rule was integrated into the protocol to facilitate interpretation of the results, as follows: HRQoL would be considered as being improved in one arm if at least one time to HRQoL score deterioration among the 5 targeted dimensions was significantly longer without a significantly shorter time to HRQoL score deterioration for the other 4 targeted dimensions.

Since AFUGEM study is a noncomparative study, *P*‐values of the treatment effect are not reported, while effect sizes are presented for exploratory purposes using HRs and 90% CIs. A 5 point difference in HRQoL scores was considered as the minimal clinically important difference.^15^

#### 2.3.2 Descriptive analysis at baseline

Quantitative variables are described using median and range. Qualitative variables are summarized using number and percentage.

The profile of missing HRQoL data at baseline was explored.^16^ Analyses were carried out by comparing 2 groups of patients: patients who completed the baseline HRQoL questionnaire (mITT population) versus those who did not. In order to determine whether baseline missing data were dependent on the patients’ characteristics, the comparison was performed according to baseline clinical and socio‐demographic variables, using the *t* test or Mann‐Whitney nonparametric test for continuous variables, and χ^2^ or Fisher's exact test for qualitative variables. *P*‐values < 0.1 were considered as significant. To determine whether baseline missing data depended on patients’ health status, the comparison was performed according to overall survival. Overall survival curves were estimated using the Kaplan‐Meier estimation method, described using median and 90% CI and compared using the log‐rank test. Univariate Cox analysis was used to estimate the HR and 90% CI.

#### 2.3.3 Longitudinal analysis

HRQoL deterioration‐free survival (QFS) was used as a modality of longitudinal analysis. QFS was defined as the time between randomization and the first HRQoL score deterioration of at least 5 points, as compared to the baseline score, with no further improvement of at least 5 points as compared to the baseline score, or death, whichever occurred first.^17^, ^18^ QFS curves were estimated using the Kaplan‐Meier estimation method. Univariate Cox models were used to calculate HRs and 90% CIs of the treatment effect. All variables collected at baseline were tested by univariate Cox analysis. The impact of time to toxicity grade 3‐4 was also tested by univariate analysis as a time‐dependent variable.

Variables significant at a threshold of 10% by univariate analyses were eligible for the multivariate model. The treatment arm was forced in the model. Restricted mean method was used as a supplement to the HR in case of nonrespect of the proportional hazards assumption in the Cox model, and also to ensure the robustness of the model. The difference of restricted mean survival time was estimated with 90% CI. A difference of restricted mean survival time greater than zero favored the sLV5FU2 group.^19^

#### 2.3.4 Sensitivity analyses

Several sensitivity analyses were performed.

First, analyses were repeated excluding death as an event in the QFS definition, which then becomes simply the time until definitive HRQoL deterioration (TUDD).^17^, ^18^

Then, QFS analyses were repeated after imputation of baseline missing data in order to consider all ITT patients. Multiple imputations of baseline missing items were performed using the Markov Chain Monte Carlo method taking into account the profile of missing data. Multivariate models were then constructed introducing the same variables as for the longitudinal analysis before treatment of missing data.

Analyses were performed using SAS (version 9.3) (SAS Institute Inc, Cary, NC) and R (version 3.3.1) software.

## 3 RESULTS

### 3.1 Study population

**Figure 1:**
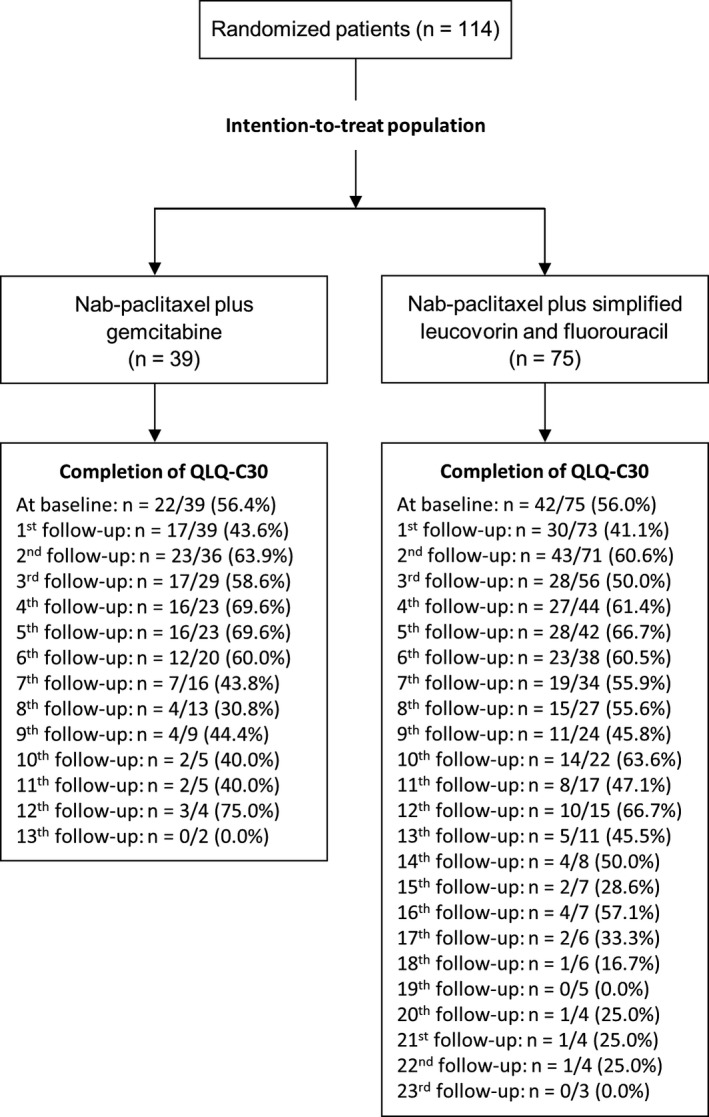
CONSORT diagram for EORTC QLQ‐C30 questionnaire

Between December 2013 and October 2014, 114 patients were randomized: 39 in the gemcitabine group and 75 in the sLV5FU2 group. Sixty‐four patients (56.1%) completed the QLQ‐C30 questionnaire at baseline, 22 patients (56.4%) in the gemcitabine group, and 42 (56.0%) in the sLV5FU2 group (Figure 1). Patients in the sLV5FU2 group completed the questionnaire longer after compared to the gemcitabine group. Indeed, there were more patients included in this treatment arm due to the ratio 1:2 and the median overall survival was significantly longer in this treatment arm. The median age was 66 years (range 45‐86) and 70 patients (61.4%) were men. The baseline characteristics of the patients are described according to baseline HRQoL availability in Table 1. Baseline HRQoL level was similar between treatment arms (Table S1).

**Table 1:** Baseline characteristics of patients according to the availability of their baseline health‐related quality of life (HRQoL) questionnaire

	Baseline HRQoL available		HRQoL not available		*P*
	(n = 64)		(n = 50)		
	n	%	n	%	
Gender					
Male	38	59.4	32	64.0	0.615
Women	26	40.6	18	36.0	
Arm					
Gemcitabine	22	34.4	17	34.0	0.967
sLV5FU2	42	65.6	33	66.0	
ECOG Perormance Status					
0	24	37.5	13	26.0	0.193
1, 2	40	62.5	37	74.0	
Pain					
Yes	36	56.3	21	42.0	0.131
No	28	43.7	29	58.0	
Jaundice					
Yes	2	3.1	4	8.0	0.402^c^
No	62	96.9	46	92.0	
Ascites					
Yes	4	6.3	2	4.0	0.694^c^
No	60	93.7	48	96.0	
Hemoglobin					
Normal	39	60.9	30	60.0	0.919
Abnormal^a^	25	39.1	20	40.0	
Platelets					
Normal	52	81.3	44	88.0	0.327
Abnormal^a^	12	18.7	6	12.0	
Total bilirubin					
Normal	52	81.3	41	82.0	0.918
Abnormal^b^	12	18.7	9	18.0	
Alkaline phosphatase					
Normal	31	49.2	20	40.8	0.376
Abnormal^b^	32	50.8	29	59.2	
Aspartate aminotransferase					
Normal	51	79.7	31	62.0	0.037
Abnormal^b^	13	20.3	19	38.0	
Alanine aminotransferase					
Normal	45	70.3	33	66.0	0.623
Abnormal^b^	19	29.7	17	34.0	
Albumin					
Normal	39	60.9	36	73.5	0.162
Abnormal^a^	25	39.1	13	26.5	
Carcinoembryonic antigen					
Normal	23	38.3	20	46.5	0.407
Abnormal^b^	37	61.7	23	53.5	
Age (years)^f^	64	65.6 (47‐85)	50	66.4 (45‐86)	0.702^d^
Body mass index (kg/m^2^)^f^	64	23.0 (16‐33)	50	23.5 (16‐32)	0.610^d^
Neutrophils (/mm^3^)^f^	64	5425.0 (2592‐19704)	50	5707.0 (1624‐12168)	0.690^e^
Creatinine (μmol/L)^f^	64	66.5 (42‐135)	50	66.2 (29‐140)	0.671^e^
Gamma‐glutamyl transpeptidase (U/L)^f^	61	134.0 (14‐1564)	44	204.5 (15‐920)	0.086^e^
Cancer antigen 19‐9 (UI/L)^f^	61	475.2 (2‐481206)	44	1391.0 (5‐214000)	0.039^e^

A χ^2^ test is used unless indicated otherwise.

a abnormally low or high levels

b abnormally high level

c Fisher's exact test

d *t* test

e Mann‐Whitney nonparametric test; sLV5FU2, simplified leucovorin and fluorouracil

f Median (range) for continuous variables.

### 3.2 Missing data analysis

**Figure 2:**
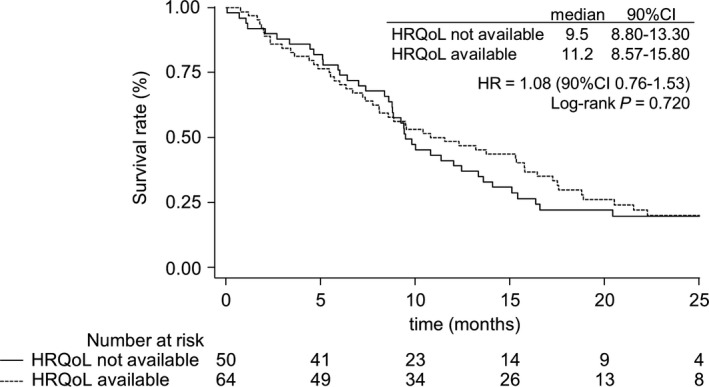
Overall survival curves according to baseline health‐related quality of life (HRQoL) availability. HR, hazard ratio; 90% CI, 90% confidence interval

Regarding the baseline characteristics, patients with available baseline HRQoL differed from other patients in terms of aspartate aminotransferase (*P* = 0.037), gamma‐glutamyl transpeptidase (*P* = 0.086) and cancer antigen 19‐9 (*P* = 0.039) (Table 1). The median overall survival was 9.5 months [90% CI 8.80‐13.30] for patients without available baseline HRQoL vs 11.2 months [90% CI 8.57‐15.80] for mITT patients (HR = 1.08, [90% CI 0.76‐1.53], *P* = 0.720) (Figure 2). Thus, we could suppose that missing data at baseline depend only on patients’ baseline characteristics.

### 3.3 Longitudinal analysis

**Figure 3:**
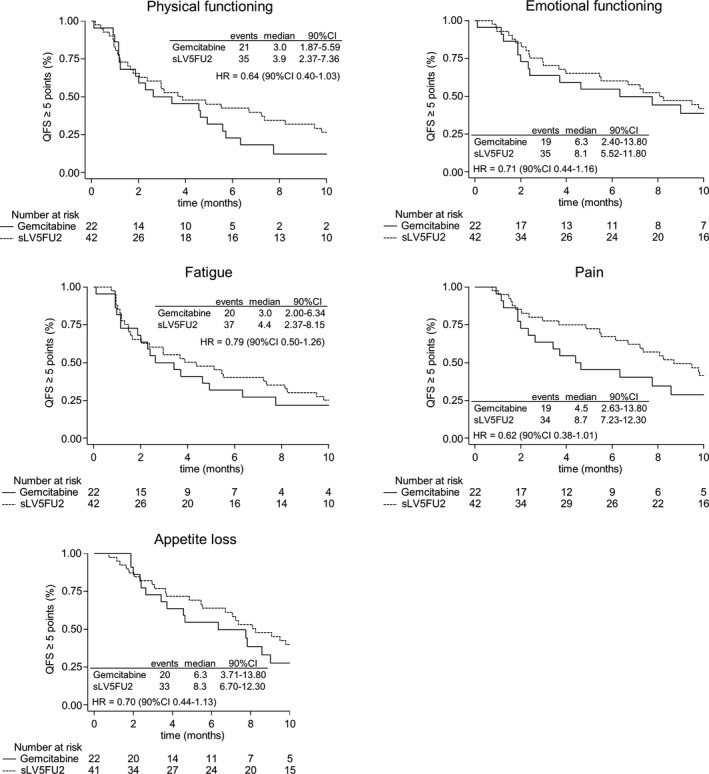
Kaplan‐Meier curves of health‐related quality of life deterioration‐free survival (QFS). HR, hazard ratio; 90% CI, 90% confidence interval

Analyses of the mITT population showed a trend towards longer QFS in favor of the sLV5FU2 group among the five targeted dimensions, namely physical functioning (HR = 0.64 [90% CI 0.40‐1.03]), emotional functioning (HR = 0.71 [90% CI 0.44‐1.16]), fatigue (HR = 0.79 [90% CI 0.50‐1.26]), pain (HR = 0.62 [90% CI 0.38‐1.01]) and appetite loss (HR = 0.70 [90% CI 0.44‐1.13]), with HRs < 1 (Figure 3). Similar trends were observed for all the other dimensions, except for constipation, which had a HR of 1.04 [90% CI 0.64‐1.69] (Table S2).

Multivariate analyses showed significantly shorter QFS among patients with the following (Table S3):

- An abnormally low level of albumin for emotional functioning (HR = 2.50 [90% CI 1.49‐4.18]) and appetite loss (HR = 2.59 [90% CI 1.50‐4.46]),
- other symptoms for fatigue (HR = 1.75 [90% CI 1.01‐3.06]),
- abnormally low level of hemoglobin for pain (HR = 3.36 [90% CI 1.98‐5.72]),
- abnormally high level of aspartate aminotransferase for appetite loss (HR = 1.86 [90% CI 1.01‐3.45]).

Results also showed significantly longer QFS among patients with the following:

- An abnormally high level of carcinoembryonic antigen for physical functioning (HR = 0.50 [90% CI 0.28‐0.88]),
- creatinine level greater than 66.2 μmol/L for fatigue (HR = 0.61 [90% CI 0.38‐0.98]),
- included in the sLV5FU2 group for pain (HR = 0.54 [90% CI 0.32‐0.92]) and appetite loss (HR = 0.55 [90% CI 0.33‐0.91]).

### 3.4 Sensitivity analysis

In the sensitivity analysis excluding death as an event, TUDD results were significantly in favor of the sLV5FU2 group for physical functioning (HR = 0.51 [90% CI 0.27‐0.97]) and pain (HR = 0.26 [90% CI 0.09‐0.74]) (Table S4). A trend toward longer TUDD in favor of the sLV5FU2 group among all other dimensions was observed, except for constipation.

After imputation of baseline missing data, results of the QFS analysis by treatment arm were similar to those obtained in the mITT population (Table S2).

Multivariate analyses after multiple imputations were in accordance with those obtained in the mITT population (Table S3). Results showed significantly shorter QFS among patients with the following:

- An abnormally high level of aspartate aminotransferase for emotional functioning (HR = 1.50 [90% CI 1.01‐2.23]),
- abnormally low level of albumin for emotional functioning (HR = 2.38 [90% CI 1.60‐3.54]) and appetite loss (HR = 2.04 [90% CI 1.38‐3.02]),
- abnormally low level of hemoglobin for fatigue (HR = 1.47 [90% CI 1.02‐2.11]) and pain (HR = 2.19 [90% CI 1.48‐3.23]),
- neutrophils level greater than 5590.0/mm^3^ for pain (HR = 1.63 [90% CI 1.14‐2.34]).

Results also showed significantly longer QFS among patients with a creatinine level greater than 66.2 μmol/L for fatigue (HR = 0.60 [90% CI 0.41‐0.89]).

## 4 DISCUSSION

In this QFS analysis performed on the population of the AFUGEM study, there was no negative impact on HRQoL in patients who received the combination of nab‐paclitaxel plus sLV5FU2. When excluding death as an event, we found that this combination improved the patients’ HRQoL level compared to gemcitabine group in terms of the decision‐rule. In fact, among the 5 targeted dimensions, TUDD was significantly longer for 2 dimensions, namely physical functioning (HR = 0.51 [90% CI 0.27‐0.97]) and pain (HR = 0.26 [90% CI 0.09‐0.74]), without a significantly shorter TUDD for the other three targeted dimensions.

In the NAPOLI‐1 phase III clinical trial in metastatic pancreatic cancer also comparing different combinations of chemotherapy, reported that liposomal irinotecan plus 5‐fluorouracil and leucovorin maintained HRQoL as assessed with the QLQ‐C30 versus 5‐fluorouracil and leucovorin while improved overall survival.^20^ However, this trial was performed in second‐line treatment and thus is not completely similar to the AFUGEM study. In another phase III trial comparing gemcitabine to the PEFG (cisplatin, epirubicin, 5‐fluorouracil, gemcitabine) regimen suggested that the combination improved overall survival while providing more grade 3‐4 neutropenia and thrombocytopenia.^21^ The impact of the treatment on HRQoL was thus important to study and the authors reported a maintained HRQoL using also the QLQ‐C30 among other questionnaires.^22^ Thus, our results are consistent with other studies using similar chemotherapy combinations.

Moreover, these results are consistent with the tolerable toxicity profile of the combination nab‐paclitaxel plus sLV5FU2 highlighted previously.^11^ These physician‐reported toxicities are thus in accordance with the patients’ perception of their own HRQoL. However, only general symptoms related to cancer were assessed using the QLQ‐C30 questionnaire and specific pancreatic cancer symptoms, such as altered bowel habits or indigestion symptoms, could not be captured. Although the EORTC pancreatic cancer module was available at the time of the study,^23^ it was not administered in this phase II trial to limit the patient burden and thus, occurrence of missing data.

The main limitation of this study was the relatively low proportion (56.1%) of questionnaires completed at baseline. However, the completion rate was similar in both treatment arms. A likely explanation could be that the first two HRQoL assessment times were at very short interval (ie, at randomization and day one of the first chemotherapy cycle) and this may have caused some confusion between these two time points at the time of data collection. The completion rate over time was close to those observed in other clinical trials in pancreatic cancer, such as in a phase II clinical trial in resectable or borderline resectable pancreatic cancer patients using also the QLQ‐C30 questionnaire to assess HRQoL among other questionnaires.^24^ These missing data also had a direct impact on the population for analysis. It was required to define a modified ITT population including all ITT patients with a baseline HRQoL score available. However, it is recommended that the ITT population be studied in order to best reflect the treatment effect without inducing bias.^14^ It is therefore essential to verify that the modified ITT is representative of the ITT population.

We addressed this issue in the sensitivity analysis, using multiple imputations. The profile of missing data was explored and was dealt only at baseline, and we hypothesized that missing data depended on the baseline characteristics of the patients themselves. Our results showed a trend toward longer QFS in favor of the sLV5FU2 group for 14 out of 15 dimensions, in analyses before and after treatment of missing data. Thus, the occurrence of missing data does not appear to have biased the results. In fact, determining the mechanism of missing data is of fundamental importance to identify the appropriate strategy for analysis of these missing data,^25^, ^26^ but this remains rare in the analysis of HRQoL in oncology clinical trials.^27^ Inadequate consideration and handling of missing data in the analysis can bias the results.^25^, ^28^ In the future, although it is a wellknown fact, it remains important to find the necessary means in the HRQoL data collection during the study to avoid missing data. HRQoL is now recognized as a key endpoint and decision criterion, and should be assessed as rigorously as other, classical biological and clinical endpoints that are required in clinical trials.

Another important point was the consideration of death in the time to HRQoL deterioration method. In this study, two approaches were adopted: first, excluding death, and second, considering death as an event within definition of time to HRQoL deterioration. Since a large number of deaths were observed, considering death as an event seemed to be the most appropriate solution and thus was retained as our main analysis. However, future research must pay greater attention to the consideration of death, for example by exploring the competing risk between death and deterioration of HRQoL.

In conclusion, this study suggests that nab‐paclitaxel plus simplified leucovorin and fluorouracil does not have a negative impact on HRQoL compared to nab‐paclitaxel plus gemcitabine. Thus, this combination of chemotherapy does not yield a clinical benefit at the cost of reduced quality of life.

## CONFLICT OF INTEREST

J‐BB has received personal fees from Amgen, Bayer, Celgene, Merck Serono, Roche, Sanofi, Servier, and nonfinancial support from Amgen, Merck Serono, and Roche. PH has received grants from Celgene and Roche; personal fees from Baxalta, Celgene, Ipsen, Lilly, Merck Serono, Novartis, and Pfizer; and nonfinancial support from Celgene, Ipsen, Merck Serono, Novartis, and Pfizer. BC has received personal fees from Bayer, Kantar Health, Kephren, Lilly, Sanofi, and nonfinancial support from Amgen, Merck Serono, and Roche. PD has received grants from DRCD Paris and personal fees from ITAC CME. J‐FS has received personal fees from Bayer, Celgene, Lilly, Novartis, Pfizer, and Servier, and nonfinancial support from Roche. JT has received personal fees from Amgen, Celgene, Baxalta, Merck Serono, Sanofi, Sirtex, and Roche. CL received personal fees from Celgene, Roche, and Sanofi. FB received grants from Novartis and Roche; personal fees from BMS, Celgene, Integragen, Ipsen, Janssen, Merck Serono, Nestle, Novartis, and Roche; and nonfinancial support from Celgene, Ipsen, Merck Serono, Novartis, and Roche. All other authors declare no conflict of interest.

## AUTHOR CONTRIBUTIONS

Emilie Charton: Formal analysis, writing – original draft, and writing – review and editing. Jean‐Baptiste Bachet: Conceptualization, data curation, funding acquisition, resources, supervision, validation, writing – original draft, and writing – review and editing. Pascal Hammel: Conceptualization, resources, and writing – review and editing. Jérôme Desramé: Resources, and writing – review and editing. Benoist Chibaudel: Resources, and writing – review and editing. Romain Cohen: Writing – review and editing. Philippe Debourdeau: Resources, and writing – review and editing. Jérome Dauba: Resources, and writing – review and editing. Thierry Lecomte: Resources, and writing – review and editing. Jean‐François Seitz: Resources, and writing – review and editing. Christophe Tournigand: Resources, and writing – review and editing. Thomas Aparicio: Resources, and writing – review and editing. Véronique Guerin‐Meyer: Resources, and writing – review and editing. Julien Taieb: Resources, and writing – review and editing. Julien Volet: Resources, and writing – review and editing. Christophe Louvet: Conceptualization, resources, and writing – review and editing. Amélie Anota: Formal analysis, supervision, validation, writing – original draft, and writing – review and editing. Franck Bonnetain: Conceptualization, methodology, resources, supervision, validation, writing – original draft, and writing – review and editing.

## Data Availability

Data are unsuitable for public deposition due to ethical and legal restrictions and are therefore available upon request with the signature of a data privacy form. To request the data, the readers may contact Prof. Jean‐Baptiste Bachet (jean-baptiste.bachet@aphp.fr).
