# Supplementary material for: Impact on health‐related quality of life deterioration‐free survival of a first‐line therapy combining nab‐paclitaxel plus either gemcitabine or simplified leucovorin and fluorouracil for patients with metastatic pancreatic cancer: Results of the randomized phase II AFUGEM GERCOR clinical trial
Source: Cancer Med. 2019 Jul 17;8(11):5079–88. doi: 10.1002/cam4.2311 (PMC6718524; doi:10.1002/cam4.2311)
Supplement: Supplementary file 1 [file CAM4-8-5079-s001.doc]

Supplementary Table S1. Description of health-related quality of life scores at baseline by treatment arm in the modified intention-to-treat and intention-to-treat population.

|  | **Nab-paclitaxel + gemcitabine (*n*=39)** | | | **Nab-paclitaxel + sLV5FU2 (*n*=75)** | | |
| --- | --- | --- | --- | --- | --- | --- |
|  | ***n*** | **median** | **range** | ***n*** | **median** | **range** |
| *mITT population* |  |  |  |  |  |  |
| **Global health status** | 22 | 50.0 | 0-83 | 42 | 50.0 | 0-83 |
| **Physical functioning** | 22 | 76.7 | 27-100 | 42 | 80.0 | 20-100 |
| **Role functioning** | 22 | 66.7 | 0-100 | 42 | 66.7 | 0-100 |
| **Emotional functioning** | 22 | 66.7 | 25-92 | 42 | 58.3 | 0-100 |
| **Cognitive functioning** | 22 | 83.3 | 0-100 | 42 | 83.3 | 0-100 |
| **Social functioning** | 22 | 83.3 | 33-100 | 41 | 83.3 | 0-100 |
| **Fatigue** | 22 | 50.0 | 0-100 | 42 | 44.4 | 0-100 |
| **Nausea and vomiting** | 22 | 0.0 | 0-100 | 42 | 0.0 | 0-100 |
| **Pain** | 22 | 41.7 | 0-100 | 42 | 66.7 | 0-100 |
| **Dyspnoea** | 22 | 0.0 | 0-67 | 42 | 0.0 | 0-100 |
| **Insomnia** | 22 | 33.3 | 0-100 | 42 | 66.7 | 0-100 |
| **Appetite loss** | 22 | 66.7 | 0-100 | 41 | 33.3 | 0-100 |
| **Constipation** | 21 | 33.3 | 0-67 | 41 | 33.3 | 0-100 |
| **Diarrhoea** | 22 | 0.0 | 0-67 | 42 | 0.0 | 0-100 |
| **Financial difficulties** | 22 | 0.0 | 0-100 | 42 | 0.0 | 0-100 |
|  |  |  |  |  |  |  |
| *ITT population* |  |  |  |  |  |  |
| **Global health status** | 39 | 51.7 | 0-87 | 75 | 50.0 | 0-92 |
| **Physical functioning** | 39 | 73.3 | 25-100 | 75 | 76.0 | 20-100 |
| **Role functioning** | 39 | 63.3 | 0-100 | 75 | 63.3 | 0-100 |
| **Emotional functioning** | 39 | 63.3 | 12-92 | 75 | 58.3 | 0-100 |
| **Cognitive functioning** | 39 | 73.3 | 0-100 | 75 | 73.3 | 0-100 |
| **Social functioning** | 39 | 70.0 | 30-100 | 75 | 70.0 | 0-100 |
| **Fatigue** | 39 | 51.1 | 0-100 | 75 | 51.1 | 0-100 |
| **Nausea and vomiting** | 39 | 13.3 | 0-100 | 75 | 16.7 | 0-100 |
| **Pain** | 39 | 46.7 | 0-100 | 75 | 50.0 | 0-100 |
| **Dyspnoea** | 39 | 20.0 | 0-73 | 75 | 13.3 | 0-100 |
| **Insomnia** | 39 | 40.0 | 0-100 | 75 | 53.3 | 0-100 |
| **Appetite loss** | 39 | 53.3 | 0-100 | 75 | 33.3 | 0-100 |
| **Constipation** | 39 | 33.3 | 0-100 | 75 | 33.3 | 0-100 |
| **Diarrhoea** | 39 | 0.0 | 0-80 | 75 | 20.0 | 0-100 |
| **Financial difficulties** | 39 | 0.0 | 0-100 | 75 | 0.0 | 0-100 |

sLV5FU2, simplified leucovorin and fluorouracil; mITT, modified intention-to-treat; ITT, intention-to-treat.

**Supplementary Table S2.** Results of the univariate Cox regression of health-related quality of life deterioration-free survival (QFS) for QLQ-C30 scores and comparison between treatment arms in the modified intention-to-treat and intention-to-treat population.

|  |  | **mITT population: QFS ≥ 5 points** | | | **ITT population: QFS ≥ 5 points** | |
| --- | --- | --- | --- | --- | --- | --- |
|  |  | ***n* (events)** | **HR (90%CI)** | **RMST (90%CI)** | ***n* (events)** | **HR (90%CI)** |
| **Global health status** | sLV5FU2 vs. Gemcitabine | 64 (55) | 0.80 (0.50-1.28) |  | 114 (98) | 0.92 (0.64-1.33) |
| **Physical functioning** | sLV5FU2 vs. Gemcitabine | 64 (56) | 0.64 (0.40-1.03) |  | 114 (96) | 0.80 (0.56-1.15) |
| **Role functioning** | sLV5FU2 vs. Gemcitabine | 64 (57) | 0.82 (0.51-1.30) |  | 114 (99) | 0.89 (0.62-1.27) |
| **Emotional functioning** | sLV5FU2 vs. Gemcitabine | 64 (54) | 0.71 (0.44-1.16) |  | 114 (94) | 0.86 (0.60-1.24) |
| **Cognitive functioning** | sLV5FU2 vs. Gemcitabine | 64 (56) | 0.79 (0.49-1.27) |  | 114 (96) | 0.87 (0.61-1.24) |
| **Social functioning** | sLV5FU2 vs. Gemcitabine | 63 (57) | 0.82 (0.51-1.30) |  | 114 (100) | 0.98 (0.68-1.42) |
| **Fatigue** | sLV5FU2 vs. Gemcitabine | 64 (57) | 0.79 (0.50-1.26) |  | 114 (99) | 0.87 (0.60-1.26) |
| **Nausea and vomiting** | sLV5FU2 vs. Gemcitabine | 64 (55) | 0.75 (0.47-1.19) |  | 114 (96) | 0.99 (0.69-1.42) |
| **Pain** | sLV5FU2 vs. Gemcitabine | 64 (53) | 0.62 (0.38-1.01) |  | 114 (93) | 0.80 (0.55-1.15) |
| **Dyspnoea** | sLV5FU2 vs. Gemcitabine | 64 (57) | 0.67 (0.42-1.07)* | 1.54 (-0.46;3.53) | 114 (101) | 0.83 (0.58-1.19) |
| **Insomnia** | sLV5FU2 vs. Gemcitabine | 64 (54) | 0.75 (0.47-1.20) |  | 114 (95) | 0.86 (0.60-1.22) |
| **Appetite loss** | sLV5FU2 vs. Gemcitabine | 63 (53) | 0.70 (0.44-1.13) |  | 114 (97) | 0.92 (0.61-1.38) |
| **Constipation** | sLV5FU2 vs. Gemcitabine | 62 (54) | 1.04 (0.64-1.69) |  | 114 (95) | 1.07 (0.72-1.58) |
| **Diarrhoea** | sLV5FU2 vs. Gemcitabine | 64 (55) | 0.79 (0.49-1.26) |  | 114 (98) | 0.94 (0.65-1.36) |
| **Financial difficulties** | sLV5FU2 vs. Gemcitabine | 64 (51) | 0.61 (0.37-1.02)* | 1.46 (-0.89;3.81) | 114 (91) | 0.77 (0.53-1.10) |

mITT, modified intention-to-treat; ITT, intention-to-treat; HR, hazard ratio; 90%CI, 90% confidence interval; RMST, restricted mean survival time; sLV5FU2, simplified leucovorin and fluorouracil. *The proportional hazards assumption of the Cox model was not respected. We used the RMST method when the hazards were not proportional.

Supplementary Table S3. Results of the multivariate Cox regression of health-related quality of life deterioration-free survival (QFS) for each targeted dimension, according to the modified intention-to-treat (mITT) and intention-to-treat (ITT) population.

|  |  | **mITT population: QFS ≥ 5 points** | | | **ITT population: QFS ≥ 5 points** | | |
| --- | --- | --- | --- | --- | --- | --- | --- |
|  |  | ***n* (events)** | **HR** | **90%CI** | ***n* (events)** | **HR** | **90%CI** |
| **Physical functioning** |  | 60 (52) |  |  | 103 (88) |  |  |
| Arm | sLV5FU2 vs. Gemcitabine |  | 0.89 | 0.50-1.58 |  | 0.84 | 0.55-1.28 |
| Carcinoembryonic antigen | Abnormal vs. Normal |  | 0.50 | 0.28-0.88 |  | 0.90 | 0.60-1.36 |
| **Emotional functioning** |  | 63 (54) |  |  | 112 (93) |  |  |
| Arm | sLV5FU2 vs. Gemcitabine |  | 0.62 | 0.37-1.03 |  | 0.81 | 0.55-1.19 |
| Aspartate aminotransferase | Abnormal vs. Normal |  | 1.74 | 0.96-3.18 |  | 1.50 | 1.01-2.23 |
| Albumin | Abnormal vs. Normal |  | 2.50 | 1.49-4.18 |  | 2.38 | 1.60-3.54 |
| **Fatigue** |  | 63 (56) |  |  | 113 (98) |  |  |
| Arm | sLV5FU2 vs. Gemcitabine |  | 0.90 | 0.54-1.50 |  | 0.96 | 0.65-1.42 |
| Other symptoms | Yes vs. No |  | 1.75 | 1.01-3.06 |  | 1.01 | 0.64-1.60 |
| Hemoglobin | Abnormal vs. Normal |  | 1.64 | 0.99-2.71 |  | 1.47 | 1.02-2.11 |
| Creatinine (μmol/L) | (≥ 66.2) vs. < 66.2 |  | 0.61 | 0.38-0.98 |  | 0.60 | 0.41-0.89 |
| Time to toxicity grade 3/4 | Time-dependent variable |  | 1.45 | 0.87-2.42 |  | 1.17 | 0.76-1.81 |
| **Pain** |  | 63 (52) |  |  | 113 (92) |  |  |
| Arm | sLV5FU2 vs. Gemcitabine |  | 0.54 | 0.32-0.92 |  | 0.83 | 0.58-1.21 |
| Neutrophils (/mm³) | (≥ 5590.0) vs. < 5590.0 |  | 1.38 | 0.85-2.24 |  | 1.63 | 1.14-2.34 |
| Hemoglobin | Abnormal vs. Normal |  | 3.36 | 1.98-5.72 |  | 2.19 | 1.48-3.23 |
| Time to toxicity grade 3-4 | Time-dependent variable |  | 1.58 | 0.88-2.82 |  | 1.06 | 0.67-1.67 |
| **Appetite loss** |  | 62 (52) |  |  | 112 (95) |  |  |
| Arm | sLV5FU2 vs. Gemcitabine |  | 0.55 | 0.33-0.91 |  | 0.86 | 0.57-1.32 |
| Body mass index (kg/m²) | (≥ 23.0) vs. < 23.0 |  | 0.62 | 0.38-1.04 |  | 0.73 | 0.50-1.07 |
| Aspartate aminotransferase | Abnormal vs. Normal |  | 1.86 | 1.01-3.45 |  | 1.34 | 0.90-2.00 |
| Albumin | Abnormal vs. Normal |  | 2.59 | 1.50-4.46 |  | 2.04 | 1.38-3.02 |

A patient with an abnormally high level of albumin and a patient with an abnormally high level of hemoglobin were excluded from these analyses since a high level had no negative impact on the health-related quality of life. HR, hazard ratio; 90%CI, 90% confidence interval; sLV5FU2, simplified leucovorin and fluorouracil; Other symptoms, such as anemia, anorexia, anxiety, asthenia, constipation, diarrhoea, dyspnoea, dysphagia, fever of 39 degrees, nausea, pulmonary embolism, recent diabetes, splenic vein thrombosis, vomiting and weight loss.

**Supplementary Table S4.** Results of the univariate Cox regression of time until definitive deterioration (TUDD) for QLQ-C30 scores and comparison between treatment arms in the modified intention-to-treat population.

|  |  | **TUDD ≥ 5 points** | | | |
| --- | --- | --- | --- | --- | --- |
|  |  | ***n* (events)** | **median in months (90%CI)** | **HR (90%CI)** | **RMST (90%CI)** |
| **Global health status** | Gemcitabine arm | 22 (7) | 4.7 (2.40-NA) | 1 |  |
|  | sLV5FU2 arm | 42 (14) | 8.7 (4.83-NA) | 0.83 (0.38-1.79) |  |
| **Physical functioning** | Gemcitabine arm | 22 (13) | 2.6 (1.18-NA) | 1 |  |
|  | sLV5FU2 arm | 42 (14) | NA (2.96-NA) | 0.51 (0.27-0.97)* | 0.32 (-0.36;1.01) |
| **Role functioning** | Gemcitabine arm | 22 (11) | 2.6 (2.20-NA) | 1 |  |
|  | sLV5FU2 arm | 42 (16) | 10.2 (2.53-NA) | 0.68 (0.35-1.32) |  |
| **Emotional functioning** | Gemcitabine arm | 22 (6) | NA (3.71-NA) | 1 |  |
|  | sLV5FU2 arm | 42 (7) | NA (10.22-NA) | 0.51 (0.20-1.29) |  |
| **Cognitive functioning** | Gemcitabine arm | 22 (8) | 3.7 (3.71-NA) | 1 |  |
|  | sLV5FU2 arm | 42 (12) | NA (4.37-NA) | 0.67 (0.31-1.43) |  |
| **Social functioning** | Gemcitabine arm | 22 (13) | 2.6 (1.22-5.59) | 1 |  |
|  | sLV5FU2 arm | 41 (17) | 4.8 (1.87-NA) | 0.63 (0.34-1.17) |  |
| **Fatigue** | Gemcitabine arm | 22 (11) | 2.6 (1.15-NA) | 1 |  |
|  | sLV5FU2 arm | 42 (16) | 5.1 (2.96-NA) | 0.60 (0.31-1.15) |  |
| **Nausea and vomiting** | Gemcitabine arm | 22 (8) | 7.8 (2.79-NA) | 1 |  |
|  | sLV5FU2 arm | 42 (11) | NA (6.28-NA) | 0.65 (0.30-1.40) |  |
| **Pain** | Gemcitabine arm | 22 (6) | NA (2.63-NA) | 1 |  |
|  | sLV5FU2 arm | 42 (5) | NA (9.82-NA) | 0.26 (0.09-0.74) |  |
| **Dyspnoea** | Gemcitabine arm | 22 (12) | 3.7 (2.79-NA) | 1 |  |
|  | sLV5FU2 arm | 42 (15) | 4.8 (3.15-NA) | 0.60 (0.31-1.15)* | 0.92 (-0.70;2.53) |
| **Insomnia** | Gemcitabine arm | 22 (6) | NA (3.32-NA) | 1 |  |
|  | sLV5FU2 arm | 42 (6) | NA (11.14-NA) | 0.40 (0.15-1.10) |  |
| **Appetite loss** | Gemcitabine arm | 22 (6) | 7.8 (4.57-NA) | 1 |  |
|  | sLV5FU2 arm | 41 (6) | NA (NA-NA) | 0.41 (0.15-1.08) |  |
| **Constipation** | Gemcitabine arm | 21 (3) | NA (NA-NA) | 1 |  |
|  | sLV5FU2 arm | 41 (12) | 8.7 (6.44-NA) | 2.00 (0.68-5.84) |  |
| **Diarrhoea** | Gemcitabine arm | 22 (7) | 4.6 (4.01-NA) | 1 |  |
|  | sLV5FU2 arm | 42 (12) | 6.5 (3.71-NA) | 0.75 (0.34-1.66) |  |
| **Financial difficulties** | Gemcitabine arm | 22 (4) | 9.8 (9.76-NA) | 1 |  |
|  | sLV5FU2 arm | 42 (7) | NA (NA-NA) | 0.87 (0.31-2.44) |  |

90%CI, 90% confidence interval; HR, hazard ratio; RMST, restricted mean survival time; sLV5FU2, simplified leucovorin and fluorouracil; NA, not available. *The proportional hazards assumption of the Cox model was not respected. We used the RMST method when the hazards were not proportional.
